# Supplementary material for: MOF-Derived CoSe2@NiFeOOH Arrays for Efficient Oxygen Evolution Reaction
Source: Nanomaterials (Basel). 2023 Sep 22;13(19):2621. doi: 10.3390/nano13192621 (PMC10574313; doi:10.3390/nano13192621)
Supplement: Supplementary file 1 [file nanomaterials-13-02621-s001.zip › nanomaterials-2606723-supplementary.pdf]

MOF-derived $CoSe_2$@NiFeOOH arrays for efficient oxygen evolution reaction

Yulong Tang,[1,2#], Jiangning Li,[1,2#] Zhiyi, Lu,[2,3] Yunan Wang,[2,3*] Kai Tao,[1*] Yichao Lin[2,3*]

[1]School of Materials Science & Chemical Engineering, Ningbo University, Ningbo, Zhejiang 315211, P. R. China.

[2] Ningbo Institute of Materials Technology and Engineering, Chinese Academy of Sciences, Ningbo, Zhejiang 315201, P. R. China

[3] University of Chinese Academy of Sciences, Beijing 100049, P.R. China

#These authors contribute equally

E-mails: yclin@nimte.ac.cn, wangyunan@nimte.ac.cn, taokai@nbu.edu.cn

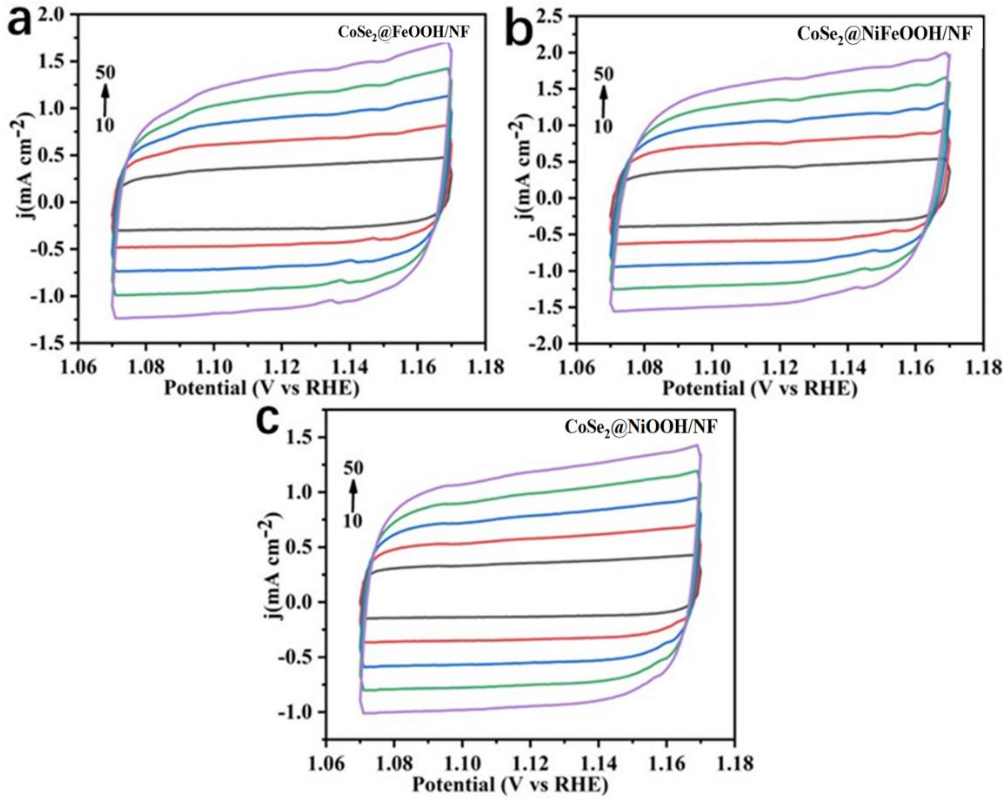

Figure S1. CV curves of a) $CoSe_2$@FeOOH/NF. b) $CoSe_2$@NiFeOOH/NF and c) $CoSe_2$@NiOOH/NF.

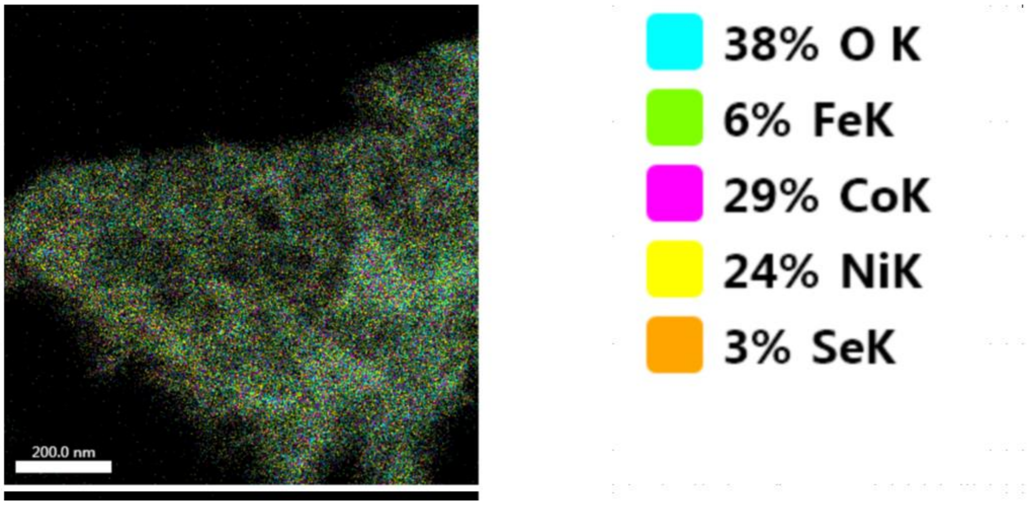

Figure S2. Elemental mapping images of Co, O, Fe, Ni and Se.

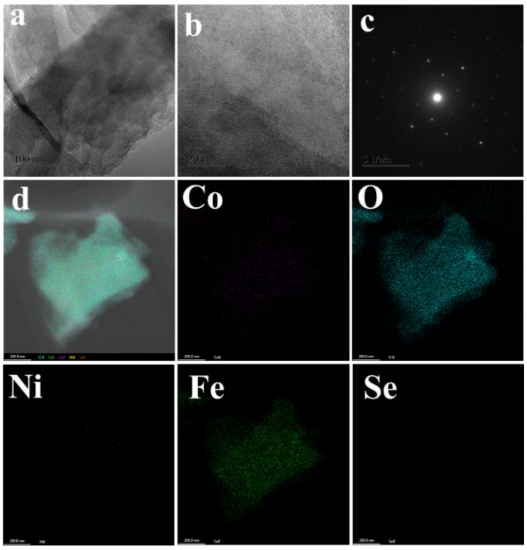

Figure S3. (a) TEM, (b) HRTEM, (c) SAED pattern, and (d) STEM images and the corresponding elemental mapping images of Co, O, Fe, Ni and Se of $CoSe_2$@NiFeOOH/NF after 100 h durability test.

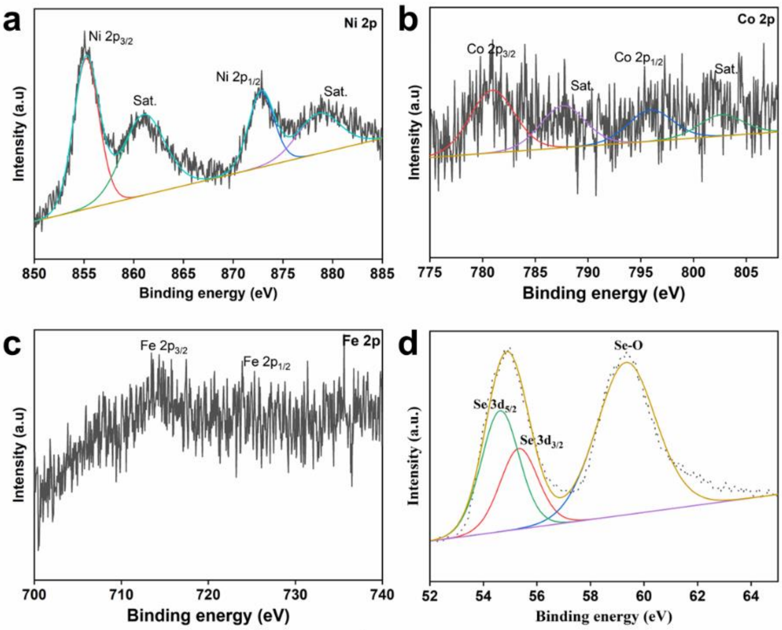

Figure S4. High-resolution XPS spectra of $CoSe_2$@NiFeOOH/NF after 100 h durability test.

Table S1. Comparison of the OER activity of $CoSe_2$@NiFeOOH/NF with other non-noble metal electrocatalysts in alkaline solution.

Electrocatalyst	j ($mA \cdot cm^{-2}$)	η(mV)	b ($mV \cdot dec^{-1}$)	Electrolyte solution	Ref.
$CoSe_2$@ NiFeOOH/NF	100	254	73	1M KOH	This work
NiFe-LDH/$NiCo_2O_4$/NF	50	350	53	1M KOH	[1]
$NiCo_2S_4$@NiFe-LDH/NF	60	201	46.3	1M KOH	[2]
NiFe:Pi/NiFe-LDH/CFP	10	290	38	1M KOH	[3]
CoSe/NiFe-LDH/	150	270	57	1M KOH	[3]
NiFe LDH	10	300	40	1M KOH	[4]
NiFe LDH/RGO	10	245	N/A	1M KOH	[5]
Ni-Fe LDH/3D-ErGO	10	259	39	1M KOH	[6]
NiFe-LDH	10	290	51	1M KOH	[7]
NiFeCo LDH/CF	10	249	42	1M KOH	[8]
Co_3O_4@NiFe-LDH	10	215	40.4	1M KOH	[9]
Mo-Ni_2P/NiFe LDH/NF	40	269	44	1M KOH	[10]
$CoSe_2$	10	284	46.3	1M KOH	[11]
$CoSe_2$-450	10	330	79	1M KOH	[12]
Co_2P/$CoSe_2$-300	100	280	51.2	1M KOH	[13]
CoSe/Ti	10	292	69	1M KOH	[14]
$NiSe_2$-$CoSe_2$	10	250	49.3	1M KOH	[15]
SiO_2/Co_xP	10	293	120	1M KOH	[16]
$Ni(OH)_2$@NiS_2	100	359	51.7	1M KOH	[17]

References

1. Wang, Z.; Zeng, S.; Liu, W.; Wang, X.; Li, Q.; Zhao, Z.; Geng, F. Coupling molecularly ultrathin sheets of NiFe-layered double hydroxide on NiCo2O4 nanowire arrays for highly efficient overall water-splitting activity. *ACS Appl. Mater. Interfaces* **2017**, *9*, 1488-1495.
2. Liu, J.; Wang, J.; Zhang, B.; Ruan, Y.; Lv, L.; Ji, X.; Xu, K.; Miao, L.; Jiang, J. Hierarchical NiCo2S4@NiFe LDH heterostructures supported on nickel foam for enhanced overall-water-splitting activity. *ACS Appl. Mater. Interfaces* **2017**, *9*, 15364-15372.
3. Wang, Y.; Yan, D.; El Hankari, S.; Zou, Y.; Wang, S. Recent progress on layered double hydroxides and their derivatives for electrocatalytic water splitting. Adv. Sci. **2018**, 5 1800064.
4. Song, F.; Hu, X. Exfoliation of layered double hydroxides for enhanced oxygen evolution catalysis. *Nat. Commun*. **2014**, *5*, 4477.
5. Youn, D. H.; Park, Y. B.; Kim, J. Y.; Magesh, G.; Jang, Y. J.; Lee, J. S. One-pot synthesis of NiFe layered double hydroxide/reduced graphene oxide composite as an efficient electrocatalyst for electrochemical and photoelectrochemical water oxidation. *J. Power Sources* **2015**, *294*, 437-443.
6. Yu, X.; Zhang, M.; Yuan, W.; Shi, G. A high-performance three-dimensional Ni–Fe layered double hydroxide/graphene electrode for water oxidation. *J. Mater. Chem. A* **2015**, *3*, 6921-6928.
7. Zhong, H.; Liu, T.; Zhang, S.; Li, D.; Tang, P.; Alonso-Vante, N.; Feng, Y. Template-free synthesis of three-dimensional NiFe-LDH hollow microsphere with enhanced OER performance in alkaline media. *J. Electrochem. Soc*. **2019**, *33*, 130-137.
8. Lin, Y.; Wang, H.; Peng, C. K.; Bu, L.; Chiang, C. L.; Tian, K.; Zhao, Y.; Zhao, J.; Lin, Y. G.; Lee, J. M.; Gao, L. Co-induced electronic optimization of hierarchical NiFe LDH for oxygen evolution. *Small*. **2020**, *16*, e2002426.
9. Meng, L.; Xuan, H.; wang, J.; Liang, X.; Li, Y.; Yang, J.; Han, P. Flower-like Co3O4@NiFe-LDH nanosheets enable high-performance bifunctionality towards both electrocatalytic HER and OER in alkaline solution. *J. Alloys Compd*. **2022**, *919*, 165877.
10. Yang, Z.; Lin, Y.; Jiao, F.; Li, J.; Wang, J.; Gong, Y. In situ growth of 3D walnut-like nano-architecture Mo-Ni2P@NiFe LDH/NF arrays for synergistically enhanced overall water splitting. *J. Energy Chem.* **2020**, *49*, 189-197.
11. Zhang, Y.; Zhang, C.; Guo, Y.; Liu, D.; Yu, Y.; Zhang, B. Selenium vacancy-rich CoSe2 ultrathin nanomeshes with abundant active sites for electrocatalytic oxygen evolution. *J. Mater. Chem. A* **2019**, *7*, 2536-2540.
12. Liu, X.; Liu, Y.; Fan, L.-Z. MOF-derived CoSe2 microspheres with hollow interiors as high-performance electrocatalysts for the enhanced oxygen evolution reaction. *J. Mater. Chem. A* **2017**, *5*, 15310-15314.
13. Du, X.; Li, J.; Tong, K.; Zhang, X. Coupling Co2P/CoSe2 heterostructure nanoarrays for boosting overall water splitting. *Dalton Trans*. **2021**, *50*, 6650-6658.
14. Liu, T.; Liu, Q.; Asiri, A. M.; Luo, Y.; Sun, X. An amorphous CoSe2 film behaves as an active and stable full water-splitting electrocatalyst under strongly alkaline conditions. *Chem. Commun*. **2015**, *51*, 16683.
15. Li, M.; Feng, LG.; NiSe2-CoSe2 with a hybrid nanorods and nanoparticles structure for efficient oxygen evolution reaction. *Chinese J. Struc. Chem.* **2022,** 41, 2201019-2201024.
16. Zeng, X.; Zhang, H.; Zhang, X.; Zhang, Q.; Chen, Y.; Yu, R.; Moskovits, M., Coupling of ultrasmall and small Co P nanoparticles confined in porous SiO2 matrix for a robust oxygen evolution reaction. *Nano Materials Science* **2022,** *4*, 393-399.
17. Xu, SJ.; Zhou, YN.; Shen, GP.; Dong, B.; Ni(OH)2 derived from NiS2 induced by reflux playing three roles for hydrogen/oxygen evolution reaction. *Chinese J. Struc. Chem.* **2022,** 41, 2208052-2208057.
